# Immobilization of a Plant Lipase from *Pachira aquatica* in Alginate and Alginate/PVA Beads

**DOI:** 10.1155/2014/738739

**Published:** 2014-04-10

**Authors:** Bárbara M. Bonine, Patricia Peres Polizelli, Gustavo O. Bonilla-Rodriguez

**Affiliations:** ^1^Department of Chemistry and Environmental Sciences, Universidade Estadual Paulista, IBILCE-UNESP, Rua Cristovão Colombo 2265, 15054-000 São José do Rio Preto, SP, Brazil; ^2^Department of Pharmacy and Biochemistry, Centro Universitário de Rio Preto, UNIRP, Rua Ivete Gabriel Atique 45, 15025-400 São José do Rio Preto, SP, Brazil

## Abstract

This study reports the immobilization of a new lipase isolated from oleaginous seeds of *Pachira aquatica*, using beads of calcium alginate (Alg) and poly(vinyl alcohol) (PVA). We evaluated the morphology, number of cycles of reuse, optimum temperature, and temperature stability of both immobilization methods compared to the free enzyme. The immobilized enzymes were more stable than the free enzyme, keeping 60% of the original activity after 4 h at 50°C. The immobilized lipase was reused several times, with activity decreasing to approximately 50% after 5 cycles. Both the free and immobilized enzymes were found to be optimally active between 30 and 40°C.

## 1. Introduction

Enzymes have a wide variety of biotechnological, biomedical, and pharmaceutical applications, enhancing the rate of numerous commercially important reactions [1].

Enzyme immobilization is a useful technique allowing enzyme reutilization, since purified enzymes are very expensive reagents to be discarded after a single use [1]. Compared to the free enzymes, immobilized enzymes typically exhibit greater stability and enzyme activity over a broader range of pH and temperatures [2, 3] and possess other advantages such as recovery yield and reusability, possibility of continuous process, rapid termination of reactions, controlled product formation, easy removal from the reaction mixture, and adaptability to various engineering designs [4–6].

Entrapment, one of the immobilization techniques, is a physical restriction of the enzyme within a confined space or network, easily obtained by gelation of polyanionic or polycationic polymers in the presence of multivalent counterions. Alginates (Alg) are one of the most used polymers due to their mild gelling properties and nontoxicity, improving enzyme stability and functional properties, as shown in trypsin [7], papain [8], invertase [9], and fungal lipases [10]. Alginate is a water-soluble anionic linear polysaccharide composed of sequential arrangements of 1,4-linked *β*-D-mannuronic acid and *α*-L-guluronic acid in different proportions in sequential arrangements, which can be precipitated by the addition of Ca^2+^ ions [11, 12], producing microspheres with good strength and flexibility [13].

Poly(vinyl alcohol) (PVA) is a synthetic, nontoxic, high-strength polymer that has been used extensively in biotechnology for enzyme and cell immobilization because of its ability to stabilize proteins and preserve their activity [14, 15]. Hydrogels of PVA and calcium Alg have found extensive applications as carrier materials for immobilizing enzymes and cells [16, 17]. The lower mass transfer resistance of PVA in comparison with Alg beads suggests that the use of Alg/PVA blends could be an interesting approach to obtaining stable microspheres for use in biotechnological processes [13].

A number of enzymes have been encapsulated in a variety of polysaccharides. Some examples include invertase [16], lactase [18], lipases [1, 19–22], and horseradish peroxidase [23].

Among hydrolases, lipases occupy a prominent position and have a wide spectrum of biotechnological applications. They are able to catalyze triacylglycerol hydrolysis at an oil-water interface; this reaction is reversible, and these enzymes can also catalyze the synthesis of esters and transesterification in microaqueous conditions. Lipases found in oilseeds have a great potential for commercial exploitation in industrial processes [24], including the synthesis of food ingredients, oil chemistry, organic synthesis, paper manufacturing, biosurfactants, cosmetics, pharmaceuticals, and the use as additives in cleaning products [25].

This study had the objective of immobilizing a lipase extracted from oleaginous seeds of* Pachira aquatica* [26, 27] by entrapment in Alg and Alg/PVA beads, describing some parameters of the immobilized lipase and comparing them to the free enzyme.

## 2. Materials and Methods

### 2.1. Materials

The lipase was purified from seeds of* Pachira aquatica *trees collected at our campus, as described elsewhere [26].

### 2.2. Entrapment of the Lipase in Calcium Alg Beads

Alginate beads were prepared using four different polymer concentrations (2, 2.5, 3, and 3.5%). Alginate, CaCl_2_, and lipase solutions were prepared in pH 8.0, 30 mmol·L^−1^ Tris buffer. A 2 mL aliquot of Alg dissolved in buffer solution was mixed with 0.5 mL of a lipase solution and this mixture was added dropwise into a 5 mL CaCl_2_ (0.025–0.2 mol·L^−1^) solution with a hypodermic syringe. Washing the untrapped enzyme was performed twice with ultrapure water (ELGA Scientific) and the beads were used for further studies. A similar method was followed for the preparation of control Alg beads, without the enzyme.

Protein concentrations in the enzyme and in the washing solutions were determined as described by Bradford [28], using bovine serum albumin (BSA) as a standard.

The amount of bound enzyme was calculated according to de Queiroz et al. [13] with modifications as follows:

(1) $$\begin{array}{c} q=\frac{\left(Ci-Cf\right)}{W}V, \end{array}$$

where *q* is the amount of the encapsulated enzyme in alginate beads (U/g); *Ci* and *Cf* are the initial and final enzymatic activities (U/mL) of the medium, respectively; *V* is the medium volume (mL), and *W* is the microspheres' mass in grams.

### 2.3. Entrapment of the Lipase in Alg/PVA Beads

Aqueous solutions of Alg and PVA were prepared separately. Alginate was prepared using four different concentrations (0.5, 1, 2, and 3%), and PVA was also prepared using four different concentrations (6, 8, 10, and 12.5%). The powdered materials were dissolved in pH 8.0, 0.03 mol·L^−1^ Tris buffer at room temperature (25°C) for Alg and at 75°C for PVA, with continuous stirring. Alginate and PVA solutions were mixed in the desired proportions at room temperature and stirred overnight. Then, the lipase solution was introduced into the Alg/PVA mixture, while stirring at 150 rpm at 4°C for 1 h. The polymer solution was then added dropwise, with a hypodermic syringe for gelation in a solution saturated with boric acid and containing 2% CaCl_2_. Washing was completed twice as before with ultrapure water and the beads were used for subsequent studies.

### 2.4. Lipase Assay

The substrate for the catalytic tests was* p-*nitrophenyl palmitate (*p-*NPP) purchased from Sigma-Aldrich. This substrate was dissolved in 1% isopropanol, 2% Triton X-100 as emulsifier, and 50 mmol·L^−1^ Tris buffer pH 8.0 [29]. For the immobilized enzyme 0.7 g of microspheres was used. A negative control without the enzyme was also studied, and the reaction mixture in each set was incubated for 60 min, stopping the reaction by removing the immobilized enzyme using a sieve. The assays were performed in triplicate.

### 2.5. Beads' Characterization

The beads' morphology was analyzed using a magnifying glass SZ2-ILST (Olympus).

### 2.6. Measurement of Temperature Optima and Stability of Free and Immobilized Lipase

The temperature at which optimum activity was achieved for the free and immobilized lipases was determined by carrying out the enzyme assay at different temperatures ranging from 30 to 60°C in 0.03 mol·L^−1^ Tris buffer at pH 8.0. The thermal stability of free and immobilized lipase was ascertained by measuring the residual activity of the enzyme exposed to the same temperatures and buffer for up to 24 hours. Activities of the samples were determined at 40°C (optimum temperature), and the assays were performed in triplicate. The Tris buffers were prepared at room temperature but their pH was calculated for the temperature of the assay, using the factor ΔpK_*a*_/°C−0.031 [30].

### 2.7. Reuse of the Immobilized Lipase

In order to test the stability of the lipase entrapped in the Ca-Alg beads, the hydrolysis reaction was repeated several times. Each incubation lasted 60 min after which the beads were separated and washed with ultrapure water. The reaction medium was then replaced with fresh medium. The activity of freshly prepared beads in the first run was defined as 100%.

### 2.8. Water Absorption by the Spheres

The spheres were lyophilized, so that water could be completely removed. Subsequently they were weighed and kept in distilled water at 25°C until the equilibrium could be reached (approximately 24 hours). Afterwards, the spheres were taken out of the water, dried in absorbent paper to remove the remaining water from the surface, and weighed again.

The water absorbed by the spheres was calculated as follows [13]:

(2) $$\begin{array}{c} wt=\frac{W_{\text{wet}}-W_{\text{dry}}}{W_{\text{wet}}}\times 100, \end{array}$$

where *wt* is the quantity of the absorbed water (%), *W*_wet_ is the weight of the spheres after being sunk (using around 10 spheres), and *W*_dry_ is the spheres' weight before water soaking.

## 3. Results and Discussion

### 3.1. Entrapment of the Lipase in Calcium Alginate Beads

Because cross-linking between alginate and Ca^2+^ ions leads to gelation, alginate and CaCl_2_ concentration are significant parameters for enzyme gel entrapment [5]. Alginate concentration was increased from 2 to 3.5% (w/v), maintaining the CaCl_2_ concentration (50 mmol·L^−1^) and the weight ratio of enzyme to Alg (E/A) 5 : 2. The best condition was found to be 3% Alg and 0.5 mmol·L^−1^ CaCl_2_ (data not shown).

As with most heterogeneous catalysts, immobilized enzymes generally experience mass transfer limitations. The size of beads in which lipase is entrapped may be one of the most important parameters of lipase immobilization. It is expected that enzymes in smaller beads will show higher catalytic activity due to reduced substrate/mass transfer resistance [5, 31, 32].

### 3.2. Entrapment of Lipase in Alg/PVA Beads

**Figure 1:**
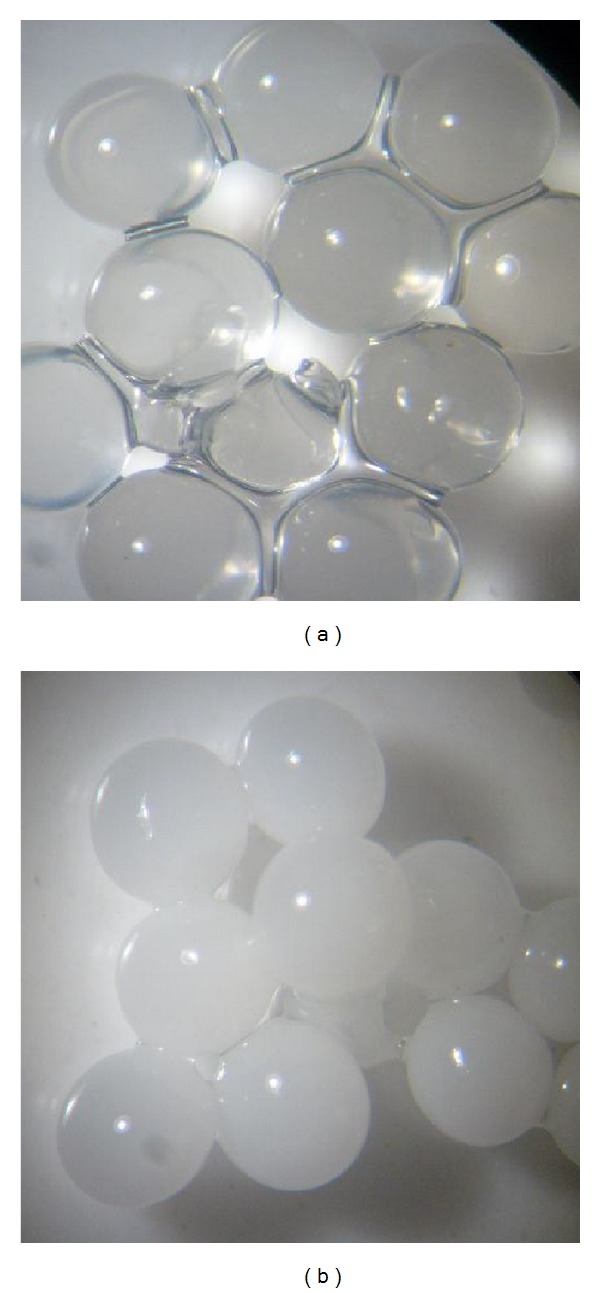
(a) Alginate spheres at 2%. (b) Alginate/PVA spheres at 12.5%. Each bead has a diameter of around 2 mm.

When associated with PVA, the best percentage of Alg was 2%. When used in this condition, the immobilized lipase could be reused up to six times and attained 23.9% of activity in the last experiment. Increases in the percentage of the sodium Alg used during the spheres' preparation reduced their aggregation (Figure 1(a)). According to Dave and Madamwar [33], sodium Alg helps the surface properties, reducing the tendency of attachment among the spheres. The condition of 2% Alg gave better separation among the spheres which enabled greater contact between the substrate and the immobilized enzyme. However, at 3%, even though the results of the spheres attachment were similar, the enzyme activity decreased quickly. Possibly this occurred because of increasing viscosity of the solution, which produces thick spheres that impair the interactions between the immobilized enzyme and the substrate.

The best percentage of PVA used associated with sodium Alg was 12.5%. This concentration allowed up to 9 consecutive utilizations, retaining 7% of the activity at the end. This result is similar to the one found in studies about the immobilization of a lipase from* Candida rugosa* in Alg-boric acid-PVA [33].  Figure 1(b) illustrates that, in this concentration (12.5%), the spheres possess a well and regular defined shape with a smooth surface.

### 3.3. Reuse of the Immobilized Lipase in Alginate and Alginate/PVA Beads

**Figure 2:**
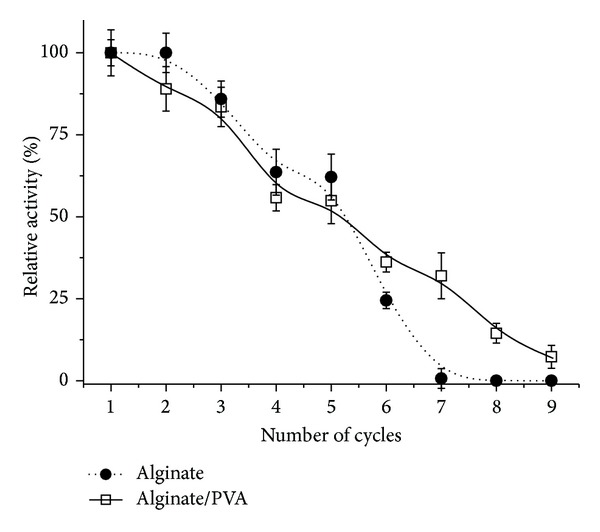
Reuse of the lipase from* Pachira aquatica* immobilized in alginate and alginate/PVA spheres. Each symbol represents the arithmetic mean of three replicates and the bars represent the standard deviation.

When comparing the performance of immobilized biocatalysts, aiming for preparative or industrial use, it is very important to characterize their operational stability, evaluated in repeated batch processes. The repeatability test was carried out under optimum conditions and the results (Figure 2) showed that the immobilized lipase in Alg could be used up to 6 cycles although with a progressive loss of activity. However, the immobilized lipase in Alg/PVA could be used additional three times, up to 9 cycles. These data are in agreement with the literature [21, 34, 35].

### 3.4. Measurement of Temperature Optima and Stability

**Figure 3:**
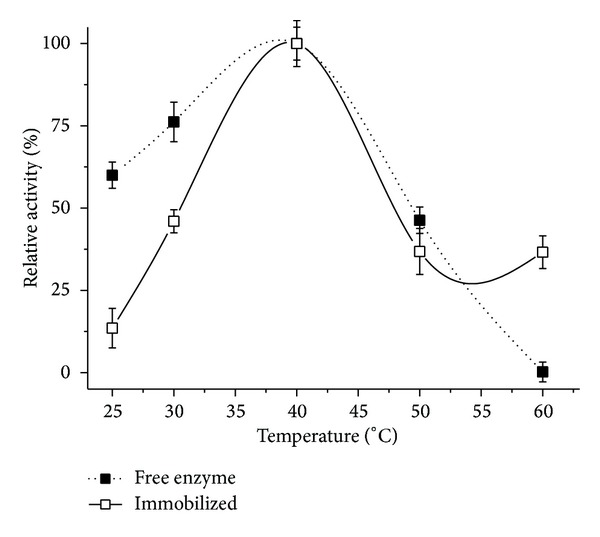
Optimum temperature for free and immobilized lipase from* Pachira aquatica*. Each symbol represents the arithmetic mean of three replicates and the bars represent the standard deviation.

Utilization of nonthermophilic enzymes in industrial processes often faces the challenge of thermal inactivation. At high temperature, the enzyme undergoes partial unfolding by heat-induced destruction of noncovalent interactions [32]. The optimum temperature for free and immobilized (Figure 3) lipase was found to be about 40°C for 90 minutes of incubation.

**Figure 4:**
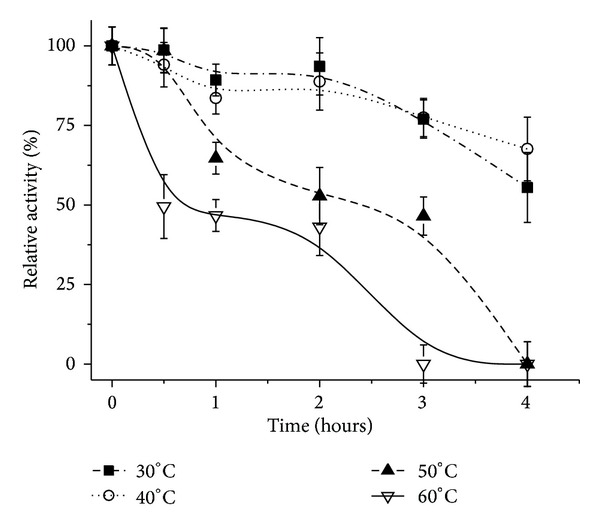
Thermal stability of the free lipase from* Pachira aquatica*. The tests were run at 37°C using* p*-nitrophenyl palmitate as substrate. Each symbol represents the arithmetic mean of three replicates and the bars represent the standard deviation.

The stability of the free enzyme at different temperatures is shown in Figure 4. For all the tested conditions, a decrease in activity occurred as the temperature increased. At lower temperatures, 30 and 40°C, the enzyme was more stable, presenting a significant decrease in activity after two hours, but still maintaining more than 50% of the activity after 4 hours. At temperature of 60°C, no activity was detected after 3 hours of exposure.

**Figure 5:**
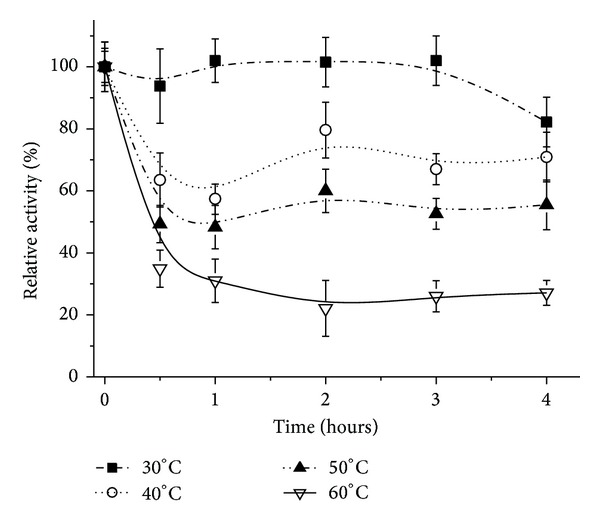
Thermal stability of the lipase from* Pachira aquatica* immobilized in calcium alginate spheres. The tests were run at 37°C using* p-*nitrophenyl palmitate as substrate. Each symbol represents the arithmetic mean of three replicates and the bars represent the standard deviation.

Figure 5 shows the stability presented for the enzyme immobilized in calcium Alg, where we observe a different behavior relative to the free enzyme. For all the tested conditions, it was verified a decrease in the activity after 1 hour of incubation, with the magnitude of the decrease being proportional to the temperature. After the initial decrease, the enzyme activity stabilized for up to 4 hours, quite apart from the continuously decreasing trend observed for the free lipase. We show that the immobilized enzyme demonstrated greater stability, as reported for pepsin immobilized in chitosan [36].

**Figure 6:**
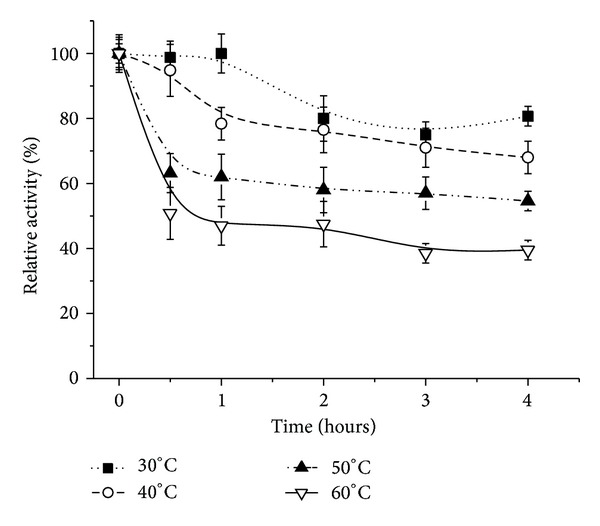
Thermal stability of the lipase from* Pachira aquatica *immobilized in spheres of PVA and alginate. The tests were run at 37°C using* p*-nitrophenyl palmitate as substrate. Each symbol represents the arithmetic mean of three replicates and the bars represent the standard deviation.

The enzyme, immobilized in calcium alginate and PVA spheres, demonstrates increased thermal stability, as is evident in Figure 6, where we see greater thermal stability, for all the tested temperatures (except 30°C), when compared with the enzyme immobilized in spheres of Alg alone or in the free form.

Lipases are extremely stable when immobilized in comparison with the free form [31], and Sharma and Gupta [32], working with several lipases, reported that Alg protected the enzymes against thermal inactivation.

The immobilization in PVA spheres would provide greater external rigidity to immobilized lipase molecules and this could reduce the effect of high temperature. Immobilized enzymes are also influenced by other factors that could contribute for their stability, such as hydrogen bonds and ionic or hidrophobic interactions [33].

### 3.5. Spheres Soaking for Alg and Alg/PVA Spheres

**Figure 7:**
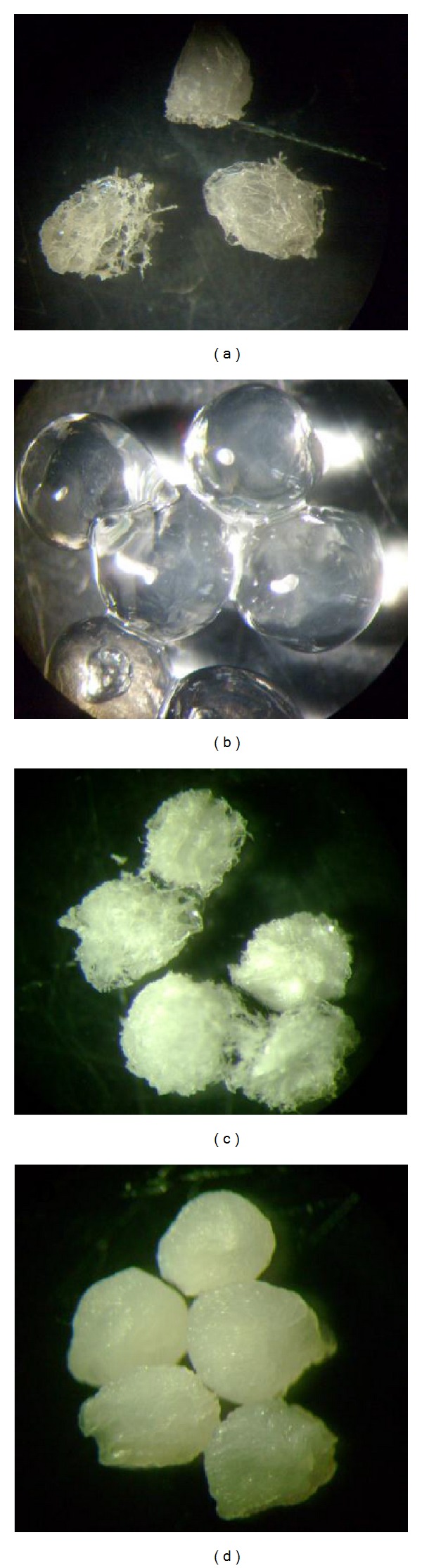
Appearance of the dry spheres and after soaking in distilled water. (a) Dried alginate spheres; (b) rehydrated alginate beads; (c) dried Alg/PVA; (d) rehydrated Alg-PVA spheres.

Figure 7 shows the morphology of the dried Alg and Alg-PVA beads, and after soaking in distilled water for 24 h.

The absorbed water by the spheres (Table 1) was calculated as proposed in Section 2.8.

The calculations showed that Alg beads absorbed approximately 3% more water when compared to the spheres of Alg and PVA. However, in both conditions, we can observe significant water absorption, which promotes an aqueous environment for lipases, reducing the problem of interfacial denaturing characteristic of biphasic systems [37]; in addition, the aqueous environment may be responsible for the catalytic difference between the immobilized lipase and the free enzyme [11, 12].

## 4. Conclusions

Immobilization of the lipase from* P. aquatica* in Alg and Alg/PVA beads improved its thermal stability in comparison to the free form and could allow its utilization in oil hydrolysis, for example.

## Figures and Tables

**Table 1:** Percentage of water absorbed in relation to the immobilization tested method. Each value represents the arithmetic mean of three replicates.

Immobilized method	% of absorbed water
Alginate	97.3 ± 0.3
Alginate and PVA	94.6 ± 1.3
